# Transcriptome Analysis of Encystation in *Entamoeba* *invadens*

**DOI:** 10.1371/journal.pone.0074840

**Published:** 2013-09-11

**Authors:** Aleyla Escueta De Cádiz, Ghulam Jeelani, Kumiko Nakada-Tsukui, Elisabet Caler, Tomoyoshi Nozaki

**Affiliations:** 1 Department of Parasitology, National Institute of Infectious Diseases, Tokyo, Japan; 2 Department of Biological Science and Environmental Studies, College of Science and Mathematics, University of the Philippines Mindanao, Davao, Philippines; 3 J. Craig Venter Institute, Rockville, Maryland, United States of America; 4 Graduate School of Life and Environmental Sciences, University of Tsukuba, Tsukuba, Ibaraki, Japan; Stanford University, United States of America

## Abstract

Encystation is an essential differentiation process for the completion of the life cycle of a group of intestinal protozoa including *Entamoeba histolytica*, the causative agent of intestinal and extraintestinal amebiasis. However, regulation of gene expression during encystation is poorly understood. To comprehensively understand the process at the molecular level, the transcriptomic profiles of *E* *. invadens* , which is a related reptilian species that causes an invasive disease similar to that of *E. histolytica*, was investigated during encystation. Using a custom-generated Affymetrix platform microarray, we performed time course (0.5, 2, 8, 24, 48, and 120 h) gene expression analysis of encysting *E* *. invadens* . ANOVA analysis revealed that a total of 1,528 genes showed ≥3 fold up-regulation at one or more time points, relative to the trophozoite stage. Of these modulated genes, 8% (116 genes) were up-regulated at the early time points (0.5, 2 and 8h), while 63% (962 genes) were up-regulated at the later time points (24, 48, and 120 h). Twenty nine percent (450 genes) are either up-regulated at 2 to 5 time points or constitutively up-regulated in both early and late stages. Among the up-regulated genes are the genes encoding transporters, cytoskeletal proteins, proteins involved in vesicular trafficking (small GTPases), Myb transcription factors, cysteine proteases, components of the proteasome, and enzymes for chitin biosynthesis. This study represents the first kinetic analysis of gene expression during differentiation from the invasive trophozoite to the dormant, infective cyst stage in *Entamoeba* . Functional analysis on individual genes and their encoded products that are modulated during encystation may lead to the discovery of targets for the development of new chemotherapeutics that interfere with stage conversion of the parasite.

## Introduction

Amebiasis is common among individuals exposed to unsanitary health conditions in developing countries. Amebiasis is also seen in developed countries among men who have sex with men and mentally handicapped people [1,2]. In both cases, the infection is established through ingestion of the cysts in feces, or fecal contaminated food and water [1]. Although *in vitro* cultivation and *in vivo* passage of the reference strains and recent clinical isolates of *E. histolytica* led to identification and characterization of the virulence mechanisms associated with amebiasis [3], the molecular mechanisms of differentiation from the invasive trophozoite to the dormant, infective cyst stage, called encystation, remains largely unknown. This is in part due to the lack of *in vitro* or *in vivo* systems that allow differentiation of *E. histolytica* [4]. To overcome this, *E* *. invadens* , which is a related reptilian species that causes an invasive disease similar to that of *E. histolytica*, has been used as a model system for encystation as *E* *. invadens* trophozoites can be induced to encyst in axenic conditions [5–7]. The morphology, the life cycle consisting of binary stages, the sites of encystation, invasiveness to the colonic epithelium, and potential dissemination from the intestine into other organs through the portal vein are similar between the two species.

Several studies focused on identifying genes involved in the stage conversion of *Entamoeba* . It has been shown that galactose/N-acetylgalactosamine, proteasome, beta- adrenergic components, and transcription factors Myb affect stage switching [4,8–12]. Protein kinase C inhibitors and short chain fatty acids have also been linked to encystation [13–15]. In *Giardia lamblia*, cysteine proteases (CP) and UDP-N-acetylglucosamine pyrophosphorylase have been shown to be key enzymes during encystation [16,17]. Similarly, an *E* *. invadens* CP isotype was found elevated in encysting cells [18]. In the social ameba *Dictyostelium discoideum*, cyclic AMP is used as an autocrine factor for sporulation [19].

Availability of the whole genome sequence of *E. histolytica* has facilitated production of custom-made DNA microarray necessary for identification and classification of genes related to virulence [20,21], the response against oxidative and nitrosative stresses [22], and stage conversion [12]. A Myb transcription factor in *E. histolytica* was also found to regulate transcription of stage-specific genes [23]. Here, we present the whole genome transcriptional profiling of *E* *. invadens* during encystation. Genes modulated during encystation and their patterns are examined to identify genes and pathways that are involved in encystation.

## Materials and Methods

### Cultivation and encystation of *E*. *invadens*

Axenic cultures of *E* *. invadens* strain IP-1 trophozoites were maintained in BI-S-33 medium at 26 °C. To induce encystation, in two independent experiments in triplicate, trophozoites were harvested in the late logarithmic phase and the cells were transferred to a seven 36 ml flasks with 47% LG medium at a final concentration of 5 x 10^5^/ml [8]. Cells were collected at seven time points: 0, 0.5, 2, 8, 24, 48, and 120 h after exposure to the encystation medium. After incubation in 47% LG medium, total numbers of cells were counted under a microscope. One portion of the cells was saved for RNA extraction and another portion was used for the differentiation of trophozoites and cysts. For the determination of cysts, the cells were resuspended in PBS containing 0.05% sarkosyl, and allowed to sit for 20 min at room temperature [24,25]. After lysed cells were stained with 0.22% trypan blue (Wako Pure Chemical Industries Ltd., Japan), intact cysts were counted and the encystation efficiency was measured by dividing the number of cysts resistant to 0.05% sarkosyl (Sigma-Aldrich, St. Louis MO, USA) with the total number of cells suspended in PBS without sarkosyl, in two independent experiments performed in triplicate.

### RNA extraction

For isolation of RNA, the cells, harvested at various time points and tested for the sarkosyl sensitivity as described above, were washed three times with 1X PBS and collected by centrifugation at 1, 500 rpm for 5 minutes after induction to wash off the encystation medium. The collected cell pellets were resuspended in 1 ml of Trizol reagent (Invitrogen, Carlsbad, CA, USA) and lysed using a Dounce homogenizer (approximately 300 strokes) until majority of the cysts were lysed as previously described [39]. The RNA concentration for each sample was measured using a Nanodrop Spectrophotometer 1000 (Thermo Scientific, Wilmington, DE, USA). RNA integrity was checked using Bio-Rad’s Experion Automated Electrophoresis System (RNA StdSens analysis kit).

### Affymetrix Microarray Hybridization

All reagents and protocols used in this study were as described in GeneChip® Expression Analysis Technical Manual (Affymetrix, Inc. Santa Clara, CA, USA). Five arrays were used for five independently isolated RNA samples corresponding to 2 biological replicates (3 arrays for the first set and 2 arrays for the second set; two sets of encystation experiments were carried out > 1 year apart in-between), were used for each time point. Using the One-Cycle cDNA synthesis kit, 5 g of total RNA was reverse transcribed using T7-Oligo (dT) primer in the first strand cDNA synthesis. After the second strand synthesis, the double-stranded cDNA template was used for *in vitro* transcription (IVT), in the presence of biotinylated nucleotides (GeneChip IVT labeling kit) to produce Biotin-labeled cRNA. Unincorporated NTPs were removed from the biotinylated cRNA (GeneChip® sample cleanup module) and then purified, quantified and fragmented. Hybridization cocktail of eukaryotic hybridization controls and fragmented, labeled cRNA (GeneChip® Hybridization, Wash and Stain Kit) were hybridized for 16 hours at 45 °C (Hybridization Oven 640, Affymetrix) to custom-generated Affymetrix platform microarray (49-7875) with probe sets consisting of 11 probe pairs each representing 12, 384 *E* *. invadens* open reading frames (Eh_Eia520620F_Ei) and 9, 327 *E. histolytica* (Eh_Eia520620F_Eh). The array chips were washed and stained (GeneChip® Hybridization, Wash and Stain Kit) with Streptavidin–phycoerythrin Biotinylated anti-streptavidin antibody using a GeneChip® Fluidics Station 450 (Affymetrix) for 1.5 hours. After washing and staining, the GeneChip® arrays were scanned using the Hewlett-Packard Affymetrix Scanner 3000.

### Analysis of microarray data

Raw probe intensities were generated by the GeneChip Operating Software (GCOS) and GeneTitan Instrument from Affymetrix. Normalized expression values for each probe set were obtained from R 2.7.0 downloaded from the BioConductor project (http://www.bioconductor.org) using robust multiarray averaging with correction for oligosequence (gcRMA). Standard correlation coefficients were calculated using GeneSpring GX 10.0.2. Reproducibility of the experiments was determined by Pearson’s correlation coefficient and confirmed by principal component analysis. Only genes that were considered ‘present’ by GCOS at least one of three arrays at any time points were used in further analysis. One-way ANOVA analysis with Tukey’s Post Hoc test was performed to extract differentially expressed genes. Gene probe sets were considered differentially expressed between time points if they had at least a 3 fold change compared against the value at 0 hour and a p-value < 0.05, calculated using Welch’s t-test, after multiple test correction by the Benjamini–Hochberg method. A post-hoc test using Tukey’s Honestly Significant Difference test was conducted to determine significant differences between samples. The data presented in this publication have been deposited in NCBIs Gene Expression Omnibus (GEO, http://www.ncbi.nlm.nih.gov/geo/) and are accessible through GEO Series accession number GSE33312.

### Annotation of Rab small GTPases and cysteine proteases

We searched the *Entamoeba* genome database using *E. histolytica* Rab small GTPases and cysteine proteases as query [3,26]. One hundred twenty one Rab and 64 EiCPs were annotated after CLUSTAL W alignment, manually edited using BioEdit and phylogenetic tree created using MEGA4 software [27]. For details, see references [28,29].

## Results and Discussion

### Kinetics of morphological differentiation

**Figure 1:**
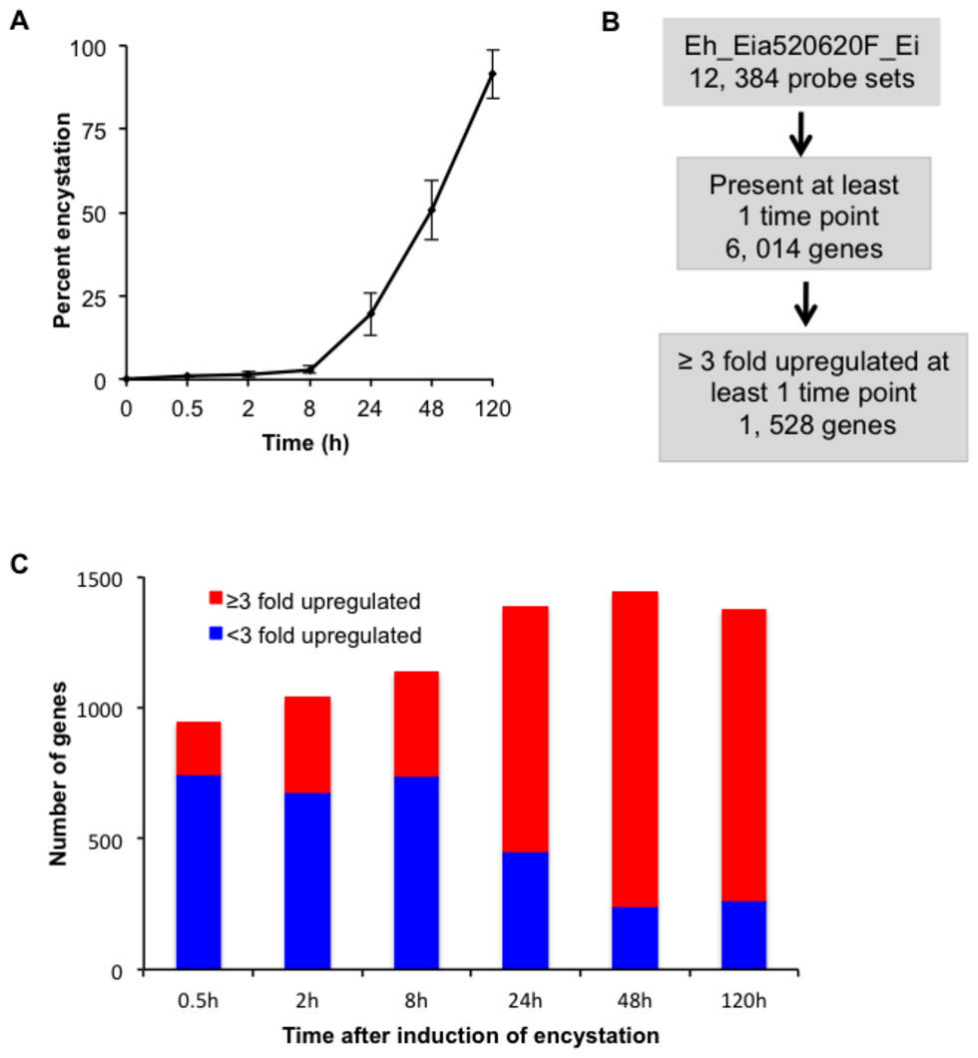
Overview of transcriptomic analysis. (**A**) **Kinetics of differentiation**. The percentages of the amoebae resistant to 0.05% sarkosyl during encystation are shown. Values are presented as % encystation and represent the mean ± S.D. of two independent experiments conducted in triplicate. (**B**) **Flow of analysis**. Microarray data were obtained in triplicates from *E* *. invadens* exposed to 47% LG medium for 0, 0.5, 2, 8, 24, 48, or 120 h, and genes expressed in at least one time point were selected for further analysis. The second data set of two biological replicates are shown as a representative. (**C**) The number of genes that were proven to be statistically significantly up-regulated by ≥3 or <3 fold at each time point of encystation.

In order to identify and characterize genes and gene cascades involved in encystation, we examined the transcriptional profiles at 7 time points (0, 0.5, 2, 8, 24, 48, and 120 h) during encystation of the reptilian ameba *E* *. invadens* . At 8 h post-induction of encystation by transferring trophozoites to the differentiation medium of low osmolarity containing no glucose, trophozoites became highly motile as compared with those maintained in the BI-S-33 medium, and only 0.9-2.8% of cysts were formed (Figure 1A). At 24 h after induction, the trophozoites rounded up, became immobile, and formed clusters, and the percentage of cysts increased to 19.7%. At 48 h of encystation, large multicellular aggregates were formed, and 50.7% of the total cells transformed into cysts. At 120h post induction 91.5% of cells transformed into cysts (Figure 1A).

### Overview of transcriptional changes during encystation

Among 12, 384 probe sets (Table S1) corresponding to *E* *. invadens* open reading frames (including 1, 272 probe sets that had been removed from NCBI), approximately 6, 014 genes were found to be expressed, i.e. had a “present” call in at least one of the five experimental replicates, at least one time point. We did not only choose genes that were found “present” in all five replicates because this will narrow down the size of genes to be analyzed during the course of encystation (Figure 1B and Table S2). These genes were filtered to extract genes whose probe sets represent a single gene (a probe set name contains suffix “_at”) or that were so highly similar in sequence to other genes as to make it impossible to design a unique probe set (a probe set with a suffix “s_at”). We set significant levels of changes to 3 fold, similar to that used in the previous work [12], where cyst-specific genes were identified in recent clinical isolates of *E. histolytica*. Furthermore, much higher numbers of genes were selected when lower fold (e.g., two fold) changes were used, which made description of modulated genes very lengthy. To validate the reproducibility of the results, we compared the transcriptomic data from the two biological replicates at different time points during encystation (Figure S1). The two data sets showed reasonable Pearson correlation coefficients (R values ranging from 0.7451 to 0.9021). We selected for the further analysis only genes that were modulated by ≥3 fold in both sets of biological replicates.

In general, the number of up- and down-regulated genes increased as encystation proceeded. We mainly focused on the up-regulated genes during encystation. The number of genes that were up-regulated ≥3 fold at any time points was 1, 528 (Table 1 and Table S3). Among the up-regulated genes, the number and proportion of the genes up-regulated ≥3 fold, compared to up-regulated < 3 fold, tends to increase at the later time points of encystation (Figure 1C) with the highest number of up-regulated genes noted at 48 h of encystation. A total of 2841 genes were down regulated by ≥3 fold at one or more time points during encystation (Table S1). At each time point, 263, 528, 543, 1325, 1835, and 1998 genes were downregulated by ≥3 fold at 0.5, 2, 8, 24, 48, and 120 h, respectively.

**Table 1:** Grouping and distribution of 1,528 genes that were up-regulated ≥3 fold at least one time points during encystation.

**Category**	**0.5h**	**2h**	**8h**	**24h**	**48h**	**120h**	**Number of genes**
1	+						11
2		+					30
3			+				10
4	+	+					26
5	+		+				1
6		+	+				15
7	+	+	+				23
8				+			85
9					+		4
10						+	57
11				+	+		89
12					+	+	358
13				+		+	7
14				+	+	+	362
15	+	+	+	+	+	+	54
16		+		+			3
17	+	+	+	+			12
18	+	+	+	+	+		13
19	+			+			3
20		+	+	+			18
21		+	+	+	+		19
22		+	+	+	+	+	81
23		+				+	2
24			+	+			16
25			+	+	+		23
26			+			+	2
27			+	+	+	+	107
28	+		+	+	+	+	4
29	+	+		+	+	+	4
30	+	+	+		+	+	1
31	+	+			+		1
32	+	+		+			3
33	+	+				+	3
34	+				+	+	10
35		+			+	+	17
36			+		+	+	6
37	+			+	+		1
38		+		+	+	+	20
39	+			+	+	+	12
40		+	+		+	+	3
41	+	+			+	+	11
42	+	+		+	+		1
			Total				1528

To identify genes that are modulated at specific time points during differentiation, we further grouped the genes into 42 categories based on expression profiles (Table 1). About 37% of genes (566) are up-regulated at 0 to 8 h of encystation; 20% (116 genes, categories 1-7) of which are exclusively up-regulated at 0 to 8 h while 80% (450 genes, categories 15-42) of those genes were also up-regulated at later time points (Table 1). List of genes up-regulated at 0.5 and/or 2 h are presented in Table S4. Genes up-regulated at 8 h are listed in Table S5.

For the 962 genes up-regulated at later time points (categories 8-14 Table 1), 9%, 0.4%, and 6% of the genes specifically peak at 24, 48 or 120 h respectively, whereas 37% (358 genes) peak at two time points (48 and 120h), 9% (89 genes) and 0.7% (7 genes) peak at 24/48 h and 24/120 h, respectively. Thirty eight percent of genes (362 genes) are continuously up-regulated at 24 to 120 h. List of genes up-regulated only at 24 h are listed in Table S6 and genes up-regulated at 48 and/or 120 h are listed in Table S7.

Only 31% (469 genes) of the up-regulated genes were annotated (Table S3). Of the 1,059 genes encoding for hypothetical proteins, 18% (187 genes) have orthologs in other organsisms. In the following sections, we summarize the modulated annotated genes based on functional classes.

### A: Bacterial surface protein A (BspA) family

**Figure 2:**
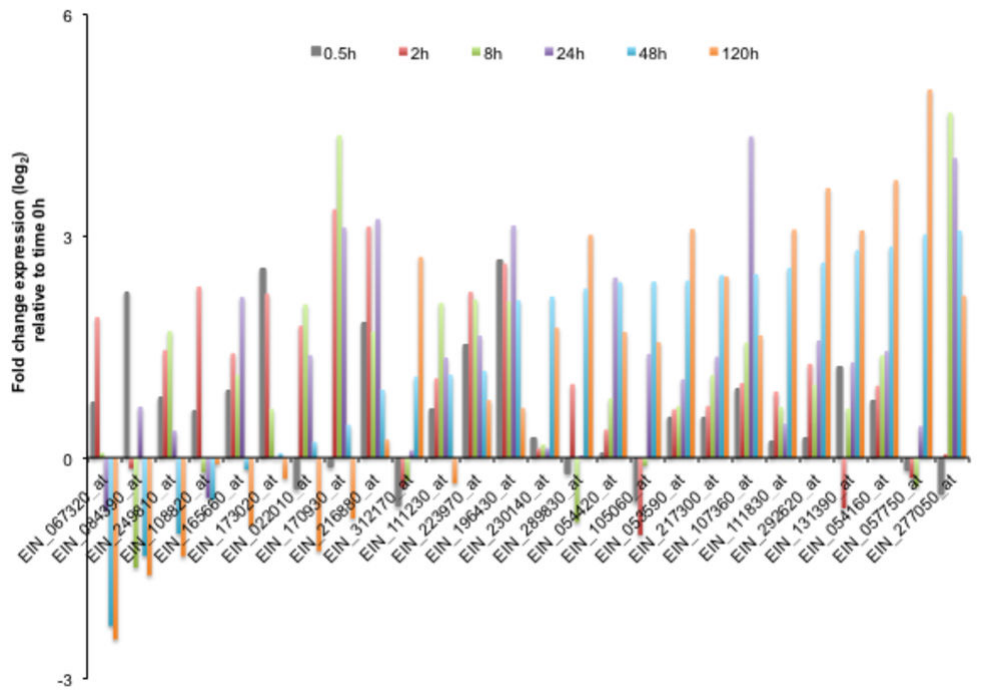
Modulation of the transcript level of the *E* *. invadens* BspA- like genes during encystation. Values are expressed as log_2_ fold change of expression relative to time 0 h.

Leucine-rich repeat (LRR)-containing proteins, which were initially identified in *Bacteroides* *forsythus* (BspA), are one of the most abundant multicopy genes in the *E* *. invadens* genome representing about 1.4% of the total *E* *. invadens* sequence reads [30]. They are annotated in AmoebaDB as hypothetical proteins with conserved regions. Similarly, 114 genes encoding for BspA-like proteins were identified in *E. histolytica* genome [31]. Homology searches using these *E. histolytica* BspA-like proteins revealed that *E* *. invadens* contain 149 BspA-like proteins (data not shown). Our transcriptome analysis showed that 26 out of 149 *E. invadens* *BspA* genes were up-regulated during encystation (Figure 2 and Table S3). About half (11) the genes were up-regulated at 48 and/or 120 h (Table S7), while 5 *BspA* genes were up-regulated at 0.5 and/or 2 h (Table S4), and the other 10 genes were up-regulated at different time points (Figure 2, Table S3). Time-dependent up-regulation of specific subsets of BspA-like genes are intriguing, as BspA was implicated in the attachment and invasion to host cells in *Treponema denticola* and *Tannerella* *forsythiae* [32,33]. One of *E. histolytica* BspA-like protein, EhLRRP1, has been shown to be localized on the cell surface, but its possible role in interaction with host ligands is not yet established [34]. As proposed in *Trichomonas vaginalis*, where BspA-like proteins might be involved in cell-cell adhesion when *T. vaginalis* forms large aggregates [35], BspA-like proteins may also be involved in a similar phenomenon in *E* *. invadens* during the early stage of encystation. Despite the similarity in the LRR repeats among *E* *. invadens* , bacteria, and trichomonads, the lack of the amino-terminal sequence and the transmembrane domain of *E* *. invadens* LRR suggests that its function is divergent [30]. Thus, up-regulation of *E* *. invadens* BspA at the late stage of encystation is highly remarkable, as BspA-like proteins were not shown associated with cell differentiation in other organisms [32,33]. It was, however, shown in *T. vaginalis* that transcript level of some BspA proteins change upon exposure in high and low iron concentrations [35]. In *E. histolytica*, iron and serum starvation resulted in the trafficking of a cytoplasmic EhRab11A protein to the cell periphery and the development of detergent resistance, similar to the cyst stage [36]. It would be interesting to show the localization of *E* *. invadens* BspA in encysting cells to determine its possible function during encystation.

### B: Cytoskeletal proteins

The Rho/Rac family of small GTP binding proteins is known to be involved in cytoskeleton regulation [37]. Two *E* *. invadens* -specific (i.e., no homolog in *E. histolytica*) *Rac* genes (EIN_166990 and EIN_017340) were modulated at early time points (Table S1), while one *RacJ* (EIN_243630) and one *RacD* (EIN_137540) genes, which also have *E. histolytica* counterparts, were up-regulated at late time points together with several Rho/Rac effectors [GTPase activating proteins (GAP) and guanine nucleotide exchange factors (GEF)]. In *E. histolytica*, it was previously shown that only a gene encoding for RacH, whose physiological role has not yet been established, was up-regulated specifically in cysts [12]. A homolog of *RacH* gene (EIN_105260) showed slight change (2 fold) in gene expression at 0.5 h of encystation (Table S1). These data are consistent with the notion that regulation of cytoskeletal rearrangement is essential at the early phase of encystation when trophozoites rearrange its surface for aggregation. It was also shown that cytochalasin D, a potent inhibitor of actin polymerization, inhibits encystation [38].

Phosphoinositides such as phosphatidylinositol 4,5-bisphosphate [PtdIns(4,5) P2] are important secondary messengers in cell surface receptor-mediated signal transduction and participate in actin cytoskeleton rearrangement [37]. The enzyme phosphatidyl 3-kinase (PI3K), which phosphorylates PtdIns, was previously shown to participate in the encystation process [13,14,39]. Our transcriptome data showed the mRNA level of one *PI3K* genes (EIN_083000) increased >3 fold at 24 to 120 h of encystation (Table S6). Furthermore, genes encoding PI(4,5) P2,3-kinase (EIN_310690) and diacylglycerol kinase (EIN_196180) were also up-regulated at later time points (Tables S6 and S7). The involvement of the pathway in encystation and excystation was previously suggested [39,40]. Recently, phosphoinositides, particularly PtdIns3P and PtdIns4P, were shown to participate in cytoskeletal rearrangement during phagocytosis of *E. histolytica* [41].

### C: Kinases and phosphatases

Tyrosine kinases play a pivotal role in sensing changes in the environment. It has been previously shown by analyzing transcriptome of recent clinical *E. histolytica* isolates that at least 14 transmembrane kinases (TMKs) are developmentally regulated [12]. Modulation of 8 TMKs were also observed *in vivo* [20]. Recently, analysis of *E. histolytica* TMK 39, 54, and 96 have shown to be involved in phagocytosis and growth [42,43]. Similarly, we identified 16 up-regulated *E* *. invadens* genes that showed significant similarity to 11 genes encoding *E. histolytica* TMKs (Table S8). Six of them were up-regulated at the early time points (*TMK 87*, Tables S4 and S5), while ten were up-regulated at the later time points (Tables S6 and S7). Forty-six *E. histolytica TMK* genes were not detected to be transcribed in trophozoites, similar to the three major cyst-specific Jacob proteins [44]. Among the *E* *. invadens* homologs corresponding to these 46 *E. histolytica TMK* genes, five *E. invadens* *TMK* gene homologs (8, 40, 38, 73, and 82) were up-regulated in later time points of encystation. However, in contrast to the previous finding, which suggested that TMK54 is involved in growth and surface expression of Gal/GalNAc lectin in trophozoites of *E. histolytica* [43], *E. invadens* *TMK 54* gene expression was not significantly modulated at early time point of encystation. These data suggest that TMK54 may have divergent functions in two species. *E. invadens* *TMK87* gene was shown to be up-regulated in *E. histolytica* in recent clinical isolates [12].

Genes encoding for serine/threonine protein phosphatases and dual specificity phosphatases were also increased during the late phase of encystation (Tables S6 and S7). In particular, 4 genes (EIN_221990, EIN_105320, EIN_020140, EIN_230540) encoding for the serine threonine phosphatases 2C (PP2C) were up-regulated during the late phase of encystation (Table S7). In yeast, PP2C phosphatases are implicated in attenuating phosphorylation during heat and osmotic shock [45]. Thus, up-regulation of PP2C might reflect anti-stress responses of *E* *. invadens* during encystation, which was reported to be induced by glucose starvation and hypo-osmotic shock [8]. However, the *E* *. invadens* PP2C homologs (Table S7) in *E. histolytica* (EHI_194220 and EHI_092510) were not shown to be cyst specific [12].

### D: Metabolism

A majority of metabolic genes involved in central energy metabolism in general were repressed. However, despite its dormant nature, genes encoding several metabolic enzymes involved in nucleotide metabolism, energy, lipids, and sphingolipids metabolism were still transcribed during the late phase of encystation (Tables S6 and S7). In our previous study we discussed detailed analysis of metabolisms of glycolysis, amino acid, cyst wall biosynthesis [46]. Briefly, among genes involved in chitin biosynthesis, the transcript level of a gene encoding for glucosamine-fructose-6-phosphate aminotransferase (GFAT, EIN_136750), which is the first and rate limiting enzyme of the chitin biosynthetic pathway, was increased at 24 h of encystation (Table S3). Chitin is the major components of the cyst wall and a homopolymer of β-1, 4-linked *N*-acetyl-glucosamine (GlcNAc) [47]. It was shown in *Giardia* that UDP-GlcNAc pyrophosphorylase (UAP) promotes the synthesis of GlcNAc and cyst wall filaments [16]. In addition, genes encoding for UDP-glucose 4-epimerase (UAE), glucosamine 6-phosphate N-acetyltransferases (GNA), phosphoglucosamine mutase (AGM), and glucosamine-6-phosphate isomerase (GNP) were also shown to be increased at mRNA and protein levels during encystation in *Giardia* [48]. However, in contrast to the findings in *Giardia* , genes for only GNA (EIN_036890) and one of UAPs (EIN_224560) were found significantly up-regulated during encystation of *E* *. invadens* (Table S3)*.*

Three genes encoding for β-1,3-N-acetylglucosaminyltransferase, involved in glycosphingolipid and glycan biosynthesis, were up-regulated at either early (EIN_068160; Table S4) or late phase (EIN_112490 and EIN_200230; Table S6) of encystation. These enzymes participate in the transfer of GlcNAc from UDP-GlcNAc onto Gal β-3 (GlcNAc β-6) GalNAc-mucin and are therefore important in chitin biosynthesis.

### E: Proteasome components, ubiquitin, and SUMO

It has been shown that expression of ubiquitin (*Ub*) gene is co-up-regulated with known cyst-specific genes during encystation. In addition, encystation was inhibited by proteasome inhibitors, suggesting that ubiquitin-proteasome activity is essential for encystation [9]. In agreement to the premise, transcription of major components of the Ub pathway such as the anaphase-promoting complex (EIN_034040), cell cycle division (EIN_192160), E2 Ub conjugating enzymes (EIN_101850), and E4 ubiquitination factor (EIN_107750) genes were up-regulated in the late phase of encystation (Tables S6 and S7). However, only *E. invadens* *Ub* gene (EIN_063840) in the *E* *. invadens* genome database was not significantly modulated during encystation process, which seems to contradict with the previous finding [9]. However, one should note that the *E. invadens* *Ub* gene previously shown to be up-regulated (AF016643 [9]) was only 52% identical to EIN_063840. The genes encoding for E1 Ub activating enzymes, 26S proteasome regulatory and core particle subunits (Table S1) were, though highly expressed, not significantly up-regulated during encystation. The gene encoding for ubiquitin carboxy-terminal hydrolase (EIN_243050) with a peptidase C19 motif was up-regulated at 8 to 120 h with a 67-fold peak expression at 24 h. A gene encoding for Ub-specific protease (EIN_107760) was also up-regulated at 24 to 48 h of encystation (Table S3). These de-ubiquitinating enzymes are likely required to process Ub-conjugated products, negatively regulate ubiquitination, and regenerate free Ub [49].

An antagonistic relationship has been established between the Ub system and sentrin/small ubiquitin-related modifier (SUMO) [50]. It has been shown that Ub and SUMO compete for a single modification site of an inhibitory protein involved in the signaling of the transcriptional activator nuclear factor–κB. SUMO-specific E2 (conjugating enzyme) Ubc9 inhibits NF-κB-dependent transcription in response to a variety of signals [50]. Our transcriptome data also showed an up-regulation of genes encoding SUMO-specific proteases (EIN_157340 and EIN_200450), SUMO ligases (EIN_168610 and EIN_081680), and Ubc9 (EIN_220240) on the late phase of encystation (Tables S6 and S7). It needs to be further determined whether Ub/SUMO antagonistic system is operated in *Entamoeba* , and the target substrates for ubiquitylation or sumoylation need to be identified [9].

### F: Protein transporters

Genes encoding for major facilitator superfamily (MFS) transporter proteins were up-regulated at different time points: EIN_257160 and EIN_040590 at 0.5 to 2 h (Table S4), EIN_054130 at 8 h (Table S5), EIN_035840 at 24 h (Table S6) and EIN_059680 at 48 to 120 h (Table S7). In general, MFS proteins facilitate the transport across the cytoplasmic or internal membranes of a variety of substrates including ions, sugar phosphates, drugs, neurotransmitters, nucleosides, amino acids, and peptides [51]. Genes encoding for two MFS (EIN_035840 and EIN_059680) that were up-regulated at the late time points were predicted to be involved in multidrug efflux as predicted by TransportDB (http://membranetransport.org/), whereas the substrates of the MFS that was expressed at the early time points (EIN_257160 and EIN_040590) were not predicted. Two genes encoding for CorA (EIN_053430 and EIN_222130) metal ion transporters (MIT), which transport magnesium/cobalt ions, were also up-regulated at 2 h (Table S4). It was previously shown that supplementation of varying concentrations and mixtures of Mg^2+^, Mn^2+^, and Co^2+^ ions to PEHPS culture medium was essential for the production of “cyst-like” structure in *E. histolytica* trophozoites [52,53]. The presence of bivalent metal ions Mn^2+^ and Co^2+^ was also shown to be necessary for augmenting chitin synthase activity in encysting *E* *. invadens* [54] and recognized as co-factors in the synthesis of the cyst wall chitin [52]. Genes encoding chitin synthases (EIN_040930 and EIN_168780) were up-regulated >3 fold starting from 2 and 8 h, respectively, and remained up-regulated up to 120 h of encystation (Table S3). The simultaneous up-regulation of metal ion transporters and chitin synthases during encystation likely supports the previous report showing that *E. histolytica* generated chitin-like material during axenic cultivation upon supplementation of these metal ions [55].

Genes encoding other ion transporters including voltage ion superfamily (EIN_036050), P-type ATPase (EIN_153520 and EIN_051610), and resistance nodulation cell division (RND) transporter (EIN_016330) were also up-regulated at the early time points (Table S4). In contrast, six transporter genes encoding the ATP-binding cassette (ABC) superfamily (EIN_015980, EIN_135600, EIN_103360, EIN_167910, EIN_146950, and EIN_059680) and four *importin* genes (EIN_219050, EIN_093910, EIN_040110, and EIN_069500) were up-regulated at the later time points (Table S7). A gene encoding for an ABC transporter (EIN_103360), previously reported to be expressed in a cyst-specific manner (EHI_178050), was also up-regulated [12]. Importin α subunit is known to bind to the nuclear localization signal of the proteins to be imported, whereas importin β subunit facilitates the docking of the importin-protein complex to the nuclear pore, respectively.

It remains still uncharacterized how the cyst wall proteins are transported in encysting trophozoites [11]. UDP-GlcNAc is the end product of the hexosamine biosynthesis pathway and the essential precursor of chitin. It was shown that accumulation of UDP-GlcNAc precedes chitin formation [46]. Two genes encoding for the UDP-GlcNAc transporter (EIN_294920 and EIN_248420) were not up-regulated with statistical significance, but gene expression slightly increased at 24 to 120 h of encystation (Table S1). These genes are known to be mainly involved in the transmembrane transport of nucleotides and sugars in the Golgi apparatus, which is the site of glycosylation, sulfation, and phosphorylation of proteoglycans and sphingolipids [56]. Up-regulation of genes encoding for UDP-GlcNAc transporters and chitinase genes (EIN_239240, EIN_053310, EIN_059870) (see below) simultaneously occurred in the late phase of encystation.

### G: Vesicular trafficking: small GTPases and their effectors

**Figure 3:**
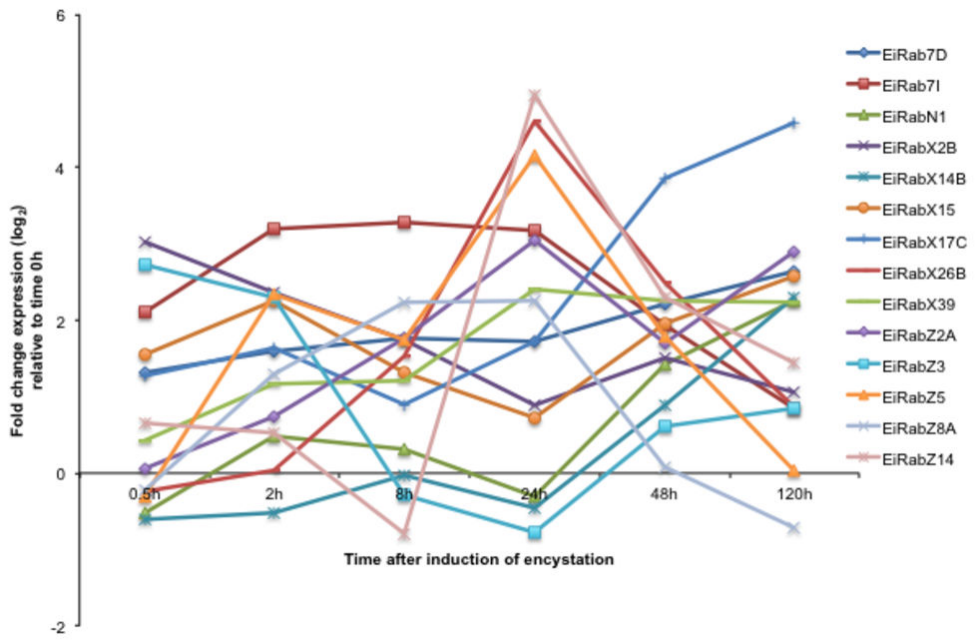
Modulation of the transcripts of 14 *E* *. invadens* Rab genes during encystation (0.5-120 h). Values are expressed as log_2_ fold change of expression relative to time 0 h. Gene IDs: EiRab7D, EIN_133760; EiRab7I, EIN_196420; EiRabN1, EIN_136950; EiRabX2B, EIN_099000; EiRabX14B, EIN_147580; EiRabX15, EIN_238750; EiRabX17C, EIN_107380; EiRabX26B, EIN_060100; EiRabX39, EIN_238590; EiRabZ2A, EIN_289320; EiRabZ3, EIN_192430; EiRabZ5, EIN_039070; EiRabZ8A, EIN_270650; EiRabZ14, EIN_061010.

*E* *. invadens* possesses 121 *Rab* genes, which were previously designated [28]. Of these 121 *Rab* genes, 14 genes (Figure 3) including 7 genes encoding RabX isotypes, which have corresponding homologs in *E. histolytica*, and 5 genes encoding *E* *. invadens* -specific (i.e., no homolog in *E. histolytica*) RabZ were up-regulated during encystation. Among the *EiRabX* isotype genes, three genes were up-regulated at later time points, one gene was up-regulated at early time points, while three other *EiRabX* isotype genes were intermittently modulated during the entire encystation process. Variation in the expression pattern of *E* *. invadens* specific *RabZ* isotype genes was also observed.

Three *E. histolytica* Rabs were previously suggested to be involved in encystation. A gene encoding for EhRab11A (previously named as EhRab11, and re-designated in reference 26) was up-regulated in a “cyst-like” form formed in a serum-deprived medium [36], while *EhRabM1* and *EhRabN1* genes were found to be highly expressed in recent clinical isolates that retained encystation ability, compared to laboratory strain, HM-1 [12]. Up-regulation of *EhRab7D* gene of an avirulent HM-1 strain was reported previously [57], but this gene was also shown to be down-regulated in recent clinical isolates [12]. Our transcriptome data showed that among two Rab subfamily (Rab7 and RabN), *EiRab7D* gene expression was up-regulated at 2 to 120 h of encystation while up-regulation of *EiRab7I* gene expression started earlier (0.5 h) and remained up-regulated up to 48 h of encystation. *EiRabN1* gene expression was upregulated at the 120 h of encystation (Figure 3).

### H: Cyst wall components

**Figure 4:**
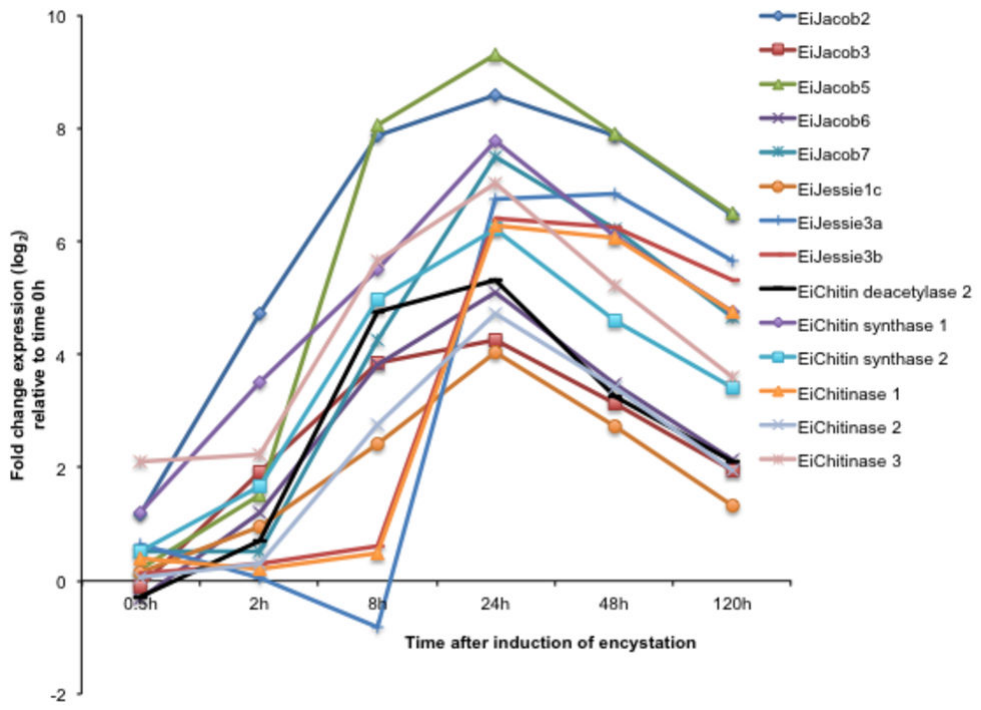
Modulation of the transcript level of the *E* *. invadens* cyst wall components during encystation (0.5-120 h). Values are expressed as log_2_ fold change of expression relative to time 0 h. Gene IDs: EiJacob 2, EIN_137570; EiJacob 3, EIN_016240; EiJacob 5, EIN_104770; EiJacob 6, EIN_015880; EiJacob 7, EIN_186850; EiJessie 1c, EIN_243430; EiJessie 3a, EIN_040990; EiJessie 3b, EIN_058620; EiChitinase 1, EIN_239240; Eichitinase 2, EIN_053310; Eichitinase 3, EIN_059870; EiChitin deacetylase 2, EIN_058630; EiChitin synthase 1, EIN_040930; EiChitin synthase 2, EIN_168780.

The major components of *E* *. invadens* cyst wall are the Jacob, Jessie lectins, and chitinase [58]. It has been proposed that these components are assembled in the cyst wall in a “wattle and daub” model, in which Jacob lectins form the wattle, chitinases cross-link the microfibrils, and Jessie lectins form the mortar or daub [59]. Chitin deacetylase acts by cleaving chitin to form chitosan on the surface of the cyst wall [47]. Based on our transcriptomic data (Figure 4), the expression patterns of each lectin and chitin subtype varied. Two genes encoding for EiJacob 1 (EIN_050710_s_at) and 4 (EIN_294450_at) were up-regulated, but the upregulation was not statistically significant. However, expression of *EiJacob1* gene increased at 0.2-120 h, while expression of *EiJacob4* gene increased from 0.5 to 48 h (Table S1), expression of genes encoding EiJacob 2, 3, and chitin synthases were up-regulated at 2 h, followed by those encoding EiJacob 5, 6, 7, chitin deacetylase 2, and chitinase 2 at 8 h. Expression of *EiJessie1c* gene was up-regulated at 8-48 h. Expression of *EiJessie3a, 3b*, and *chitinase 1* genes were upregulated at 24 h (Figure 4). Chitinase 3 was constitutively expressed. Therefore, the expression profiles of these components do not support the proposed “wattle and daub” model, and may suggest post-transcriptional regulation of these proteins.

### I. Myb transcription factors with R2R3 and ^T^/ _S_HAQK^Y^/_F_ motifs

**Figure 5:**
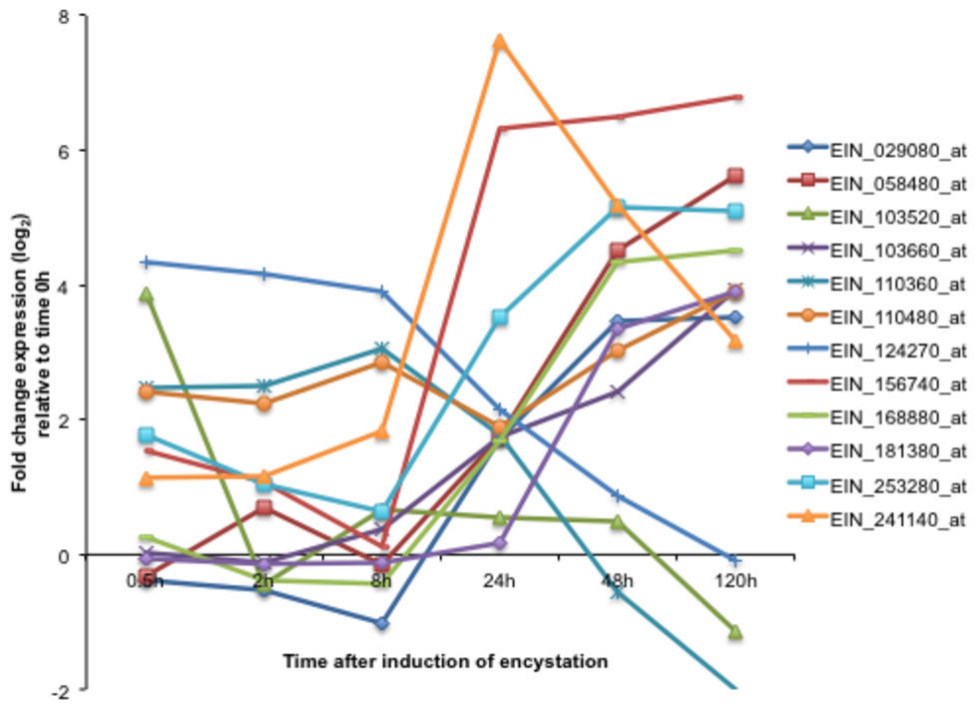
Modulation of the transcript level of the *E* *. invadens* Myb transcription factors during encystation (0.5-120 h). Values are expressed as log_2_ fold change of expression relative to time 0 h after induction of encystation.

The Myb family of transcription factors is essential in regulating cell differentiation, cell proliferation, and cell cycle [60]. Recent *in silico* analysis of the *E. histolytica* genome showed 34 proteins with Myb DNA-binding domains [61]. A gene encoding for one of these Mybs with a ^T^/ _S_HAQK^Y^/_F_ motif was shown to be developmentally regulated [12]. It has also been shown that this Myb transcription factor regulates expression of a subset of stage-specific genes in *E. histolytica* [23]. Using the Pathema *Entamoeba* genome database, we searched for *E* *. invadens* Mybs, and identified a total of 37 *E* *. invadens* Myb genes encoding Myb proteins with R2R3 and ^T^/ _S_HAQK^Y^/_F_ conserved motifs. Twenty-eight putative *E* *. invadens* Myb proteins (25 annotated and 3 hypothetical proteins) possess conserved R2R3 repeats, eleven of these were differentially expressed (Figure 5). The ^T^/ _S_HAQK^Y^/_F_ motif was found in 9 hypothetical proteins; and only one gene (EIN_241140_at) of them was up-regulated during encystation (Figure 5). The expression profiles of most of these *Myb* genes were different, which is consistent with a notion that each Myb transcription factor controls expression of specific subsets of genes on a specific phase of encystation, as previously suggested [23].

### J: Cysteine proteases

Fifty cysteine proteases (CPs) have been identified in the *E. histolytica* genome [24]. Ten of these CPs were detected in trophozoites, most of which appear to be linked with virulence [3,26,62–65]. However, the role of CPs in encystation has not been elucidated. Recently, 8 CP genes have shown to be differentially expressed in cysts using xenic *E. histolytica* clinical isolates [12].

**Figure 6:**
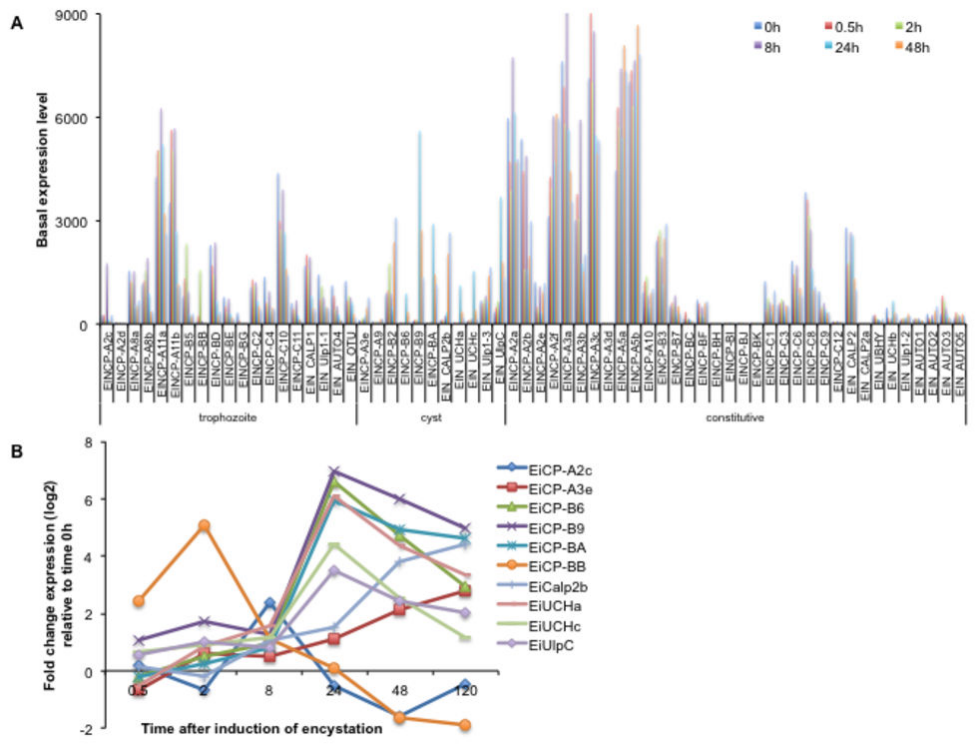
Modulation of the transcript level of the *E* *. invadens* cysteine proteases during encystation (0.5-120 h). (**A**) Sixty four CPs were grouped into trophozoite-, cyst-predominant, and constitutively expressed CPs based on the transcriptome profiles. **B**) Line graphs showing the fold change expression (log_2_) relative to time 0h of ten EiCPs whose expression were significantly modulated during encystation. Gene IDs: EiCP-A2c, EIN_168460; EiCP-A3e, EIN_192250; EiCP-B6, EIN_292720; EiCP-B9, EIN_152250; EiCP-BA, EIN_184830; EiCP-BB, EIN_199850; EiCalp2b, EIN_187000; EiUCHa, EIN_243050; EiUCHc, EIN_107760; EiUlpC, EIN_200450.

Cysteine proteases in *E* *. invadens* were previously identified and annotated [29]. We grouped 64 *E* *. invadens* CPs into three categories: cyst specific CPs (11 EiCPs), expression of which increased at 24 to 120 h; trophozoite specific CPs (19 EiCPs), expression of which was higher at 0 to 8 h of encystation compared to 24 to 120 h; and constitutively expressed CPs (34 EiCPs) (Figure 6A). Among the modulated CPs (Figure 6B), six belongs to C1 papain superfamily clades A and B (EiCP-A2c, EiCP-A3e, EiCP-BA, EiCP-BB, EiCP-B6, and EiCP-B9), one calpain-like protease (EiCalp2b), two ubiquitin carboxy-terminal hydrolases (EiUCHa and EiUCHc), and one Ulp protease (EiUlpC). Ulp proteases are a group of peptidases that control the function of SUMO. Expression of *EiCP-A2c* and *EiCP-BB* genes was up-regulated at early time points, while that of *EiCP-B9* and *EiUCHa* genes were up-regulated at 24 and 120 h, respectively. Six EiCPs were up-regulated at 24 h or later of encystation. The most striking result was the 61-fold up-regulation of *EiCP-BA* transcript at 24h of encystation. The closest homolog of EiCP-BA is EhCP-B6, although EiCP-BA has a transmembrane domain and an ERFNIN motif similar with a cathepsin L-like enzyme [3,65]. Additionally, expression levels of *EiCP-B6* and *EiCP-UCHa* genes, which were not expressed at 0-0.5 h of encystation, dramatically increased by 99 and 68 fold at 24 h, respectively. *E. histolytica* genes homologous to *EiCP-A3e* and *EiCP-B9* genes in were also up-regulated in cysts [12]. Further studies on the cellular localization of these newly-identified stage-regulated CPs are required to determine whether *E* *. invadens* CPs may be involved in encystation, as demonstrated in *Giardia lamblia*, where cysteine protease 2 was co-transported with cyst wall protein in encystation-specific vesicles and plays a central role during encystation [17].

In addition, the EhCP-B9/EhCP112 homolog in *E* *. invadens* [29] was shown to be accumulated near the cyst wall of immature cysts and further evenly distributed in the cytosol of mature cysts. Transcription of EiCP-B9 also increased 126 fold at 24 h of encystation, which coincide the initiation of the cyst wall formation [18].

### K: Heat shock proteins

Microarray analysis of heat shock induced *E. histolytica* showed up-regulation of Gal/GalNAc lectin, cysteine proteases, and heat shock proteins such as Hsp70 (EHI_197860, EHI_199590) and Hsp90 (EHI_102270, EHI_163480) [66]. Exposure of *E* *. invadens* trophozoites to similar conditions also increased the mRNA expression of *BiP* gene (GenBank AAF64243.1) and was suggested to be partially linked with encystation based on an increased expression of *Jacob* and *chitinase* genes in *E* *. invadens* , although heat shock per se did not result in the formation of the cyst wall [67]. The closest homolog of this *E. invadens* *BiP* gene on our array is luminal binding protein 4 precursor (EIN_105260, 58% identity), which also contains the ER-retention signal motif (KDEL) required for its proper targeting to the endoplasmic reticulum [67,68]. However, the mRNA level of EIN_105260 was unchanged at 0-24 h and only slightly increased (1.4 fold) at 120 h of encystation (Table S1). Our transcriptome data did not support the premise that the expression of *BiP* and *chitinase* genes is linked or coincides in *E* *. invadens* . Up-regulation of *chitinase 1* gene expression peaked at 24 h (78-fold up-regulated). Expression of *chitinase 2* genes increased by 7 fold at as early as 8 h, and peaked at 24 h (26 fold up-regulation) of encystation. In contrast, *chitinase 3* gene was constitutively expressed (Figure 4).

## Conclusions

Our transcriptomic analysis of *E* *. invadens* revealed global changes of gene expression during encystation, and should help to identify key regulatory genes that are essential for the process. Further studies on individual genes and their encoded products that are modulated during encystation may lead to the discovery of targets for the development of new chemotherapeutics that interfere with stage conversion of the parasite.

## Supporting Information

Figure S1 **Correlation between two biological replicates.** The correlation levels of transcripts in DNA microarray analysis between first and second biological replicates at different time points during encystation is shown. The Pearson correlation coefficients were calculated using Excel (2011) workbook.
(TIF)

Table S1 **List of all probe sets representing *E* *. invadens* open reading frames.** The *E* *. invadens* probeset ID, fold change and regulation relative to time 0 h (trophozoite stage), average hybridization signal intensities (raw expression data) of five arrays at each time point, normalized log_2_ transformed value, common names, gene ID and predicted GO function are shown.
(XLSX)

Table S2 **Normalized transcriptome data of genes present at least one time points during encystation.** The *E* *. invadens* probe set ID, p-value and corrected p-value of ANOVA, post-hoc test, fold change and regulation relative to time 0 h (trophozoite stage), normalized expression levels in log_2_ scale, Pathema/AmoebaDB gene ID, common names, predicted GO function of two biological replicate are shown.
(XLSX)

Table S3 **List of genes which were up-regulated ≥ 3 fold at one or more time points during encystation.** The *E* *. invadens* probeset ID, fold change and regulation relative to time 0 h (trophozoite stage), average hybridization signal intensities (raw expression data) of five arrays at each time point, normalized log_2_ transformed value, common names, gene id and predicted GO function are shown.
(XLSX)

Table S4 **List of genes which were up-regulated ≥ 3 fold at 0.5 and 2 h of encystation.** The *E* *. invadens* probeset ID, fold change and regulation relative to time 0 h (trophozoite stage), average hybridization signal intensities (raw expression data) of five arrays at each time point, normalized log_2_ transformed value, common names, gene ID and predicted GO function are shown.
(XLSX)

Table S5 **List of genes which were up-regulated ≥ 3 at 8 h of encystation.** The *E* *. invadens* probeset ID, fold change and regulation relative to time 0h (trophozoite stage), average hybridization signal intensities (raw expression data) of five arrays at each time point, normalized log_2_ transformed value, common names, gene ID and predicted GO function are shown.
(XLSX)

Table S6 **List of genes which were up-regulated ≥ 3 fold at 24 h of encystation.** The *E* *. invadens* probeset ID, fold change and regulation relative to time 0 h (trophozoite stage), average hybridization signal intensities (raw expression data) of five arrays at each time point, normalized log_2_ transformed value, common names, gene ID and predicted GO function are shown.
(XLSX)

Table S7 **List of genes which were up-regulated ≥ 3 fold at 48 and 120h of encystation.** The *E* *. invadens* probeset ID, fold change and regulation relative to time 0h (trophozoite stage), average hybridization signal intensities (raw expression data) of five arrays at each time point, normalized log_2_ transformed value, common names, gene ID and predicted GO function are shown.
(XLSX)

Table S8 **List of *E* *. invadens* transmembrane kinases genes induced ≥ 3 fold at one or more time points during encystation.** The *E* *. invadens* probeset ID, annotation, closest homolog in *E. histolytica* TMKs (number and group), expression in previous studies [12,20,43] predicted number of transmembrane domains (TMHMM) and conserved sequence motifs [43] are shown.
(XLSX)
