# Supplementary figures and images for: Transcriptome Analysis of Encystation in Entamoeba invadens
Source: PLoS One. 2013 Sep 11;8(9):e74840. doi: 10.1371/journal.pone.0074840 (PMC3770568; doi:10.1371/journal.pone.0074840)

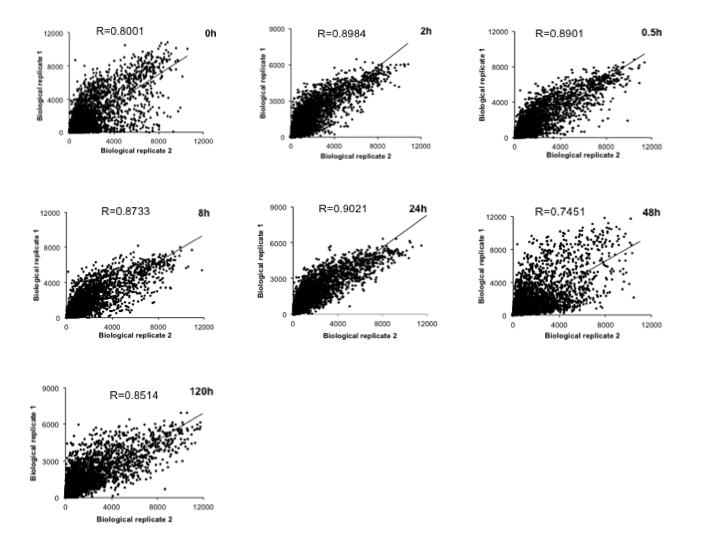

Supplement: Figure S1 — Correlation between two biological replicates. The correlation levels of transcripts in DNA microarray analysis between first and second biological replicates at different time points during encystation is shown. The Pearson correlation coefficients were calculated using Excel (2011) workbook. (TIF) [file pone.0074840.s001.tif]
